# Identifying Depressed Essential Tremor Using Resting-State Voxel-Wise Global Brain Connectivity: A Multivariate Pattern Analysis

**DOI:** 10.3389/fnhum.2021.736155

**Published:** 2021-10-12

**Authors:** Yufen Li, Li Tao, Huiyue Chen, Hansheng Wang, Xiaoyu Zhang, Xueyan Zhang, Xiyue Duan, Zhou Fang, Qin Li, Wanlin He, Fajin Lv, Jin Luo, Zheng Xiao, Jun Cao, Weidong Fang

**Affiliations:** ^1^Department of Radiology, The First Affiliated Hospital of Chongqing Medical University, Chongqing, China; ^2^Department of Neurology, The First Affiliated Hospital of Chongqing Medical University, Chongqing, China; ^3^Department of Psychiatry, The First Affiliated Hospital of Chongqing Medical University, Chongqing, China

**Keywords:** essential tremor, depression, multivariate pattern analysis, global brain connectivity, resting-state functional magnetic resonance imaging

## Abstract

**Background and Objective:** Although depression is one of the most common non-motor symptoms in essential tremor (ET), its pathogenesis and diagnosis biomarker are still unknown. Recently, machine learning multivariate pattern analysis (MVPA) combined with connectivity mapping of resting-state fMRI has provided a promising way to identify patients with depressed ET at the individual level and help to reveal the brain network pathogenesis of depression in patients with ET.

**Methods:** Based on global brain connectivity (GBC) mapping from 41 depressed ET, 49 non-depressed ET, 45 primary depression, and 43 healthy controls (HCs), multiclass Gaussian process classification (GPC) and binary support vector machine (SVM) algorithms were used to identify patients with depressed ET from non-depressed ET, primary depression, and HCs, and the accuracy and permutation tests were used to assess the classification performance.

**Results:** While the total accuracy (40.45%) of four-class GPC was poor, the four-class GPC could discriminate depressed ET from non-depressed ET, primary depression, and HCs with a sensitivity of 70.73% (*P* < 0.001). At the same time, the sensitivity of using binary SVM to discriminate depressed ET from non-depressed ET, primary depression, and HCs was 73.17, 80.49, and 75.61%, respectively (*P* < 0.001). The significant discriminative features were mainly located in cerebellar-motor-prefrontal cortex circuits (*P* < 0.001), and a further correlation analysis showed that the GBC values of significant discriminative features in the right middle prefrontal gyrus, bilateral cerebellum VI, and Crus 1 were correlated with clinical depression severity in patients with depressed ET.

**Conclusion:** Our findings demonstrated that GBC mapping combined with machine learning MVPA could be used to identify patients with depressed ET, and the GBC changes in cerebellar-prefrontal cortex circuits not only posed as the significant discriminative features but also helped to understand the network pathogenesis underlying depression in patients with ET.

## Introduction

Essential tremor (ET) has been gradually noted to contain numerous non-motor features such as depression, cognitive deficits, sleep disturbance, and anxiety, and depression is one of the most common non-motor symptoms (Chandran et al., 2012; Louis, 2016). Growing studies (Louis et al., 2016; Achey et al., 2018) pointed out that 35% of patients with ET had mild to severe depressive symptoms, and these symptoms would worsen the poor quality of life in patients with ET. However, to date, the pathogenic mechanisms in ET, let alone ET with depression are still unclear.

Using the local functional connectivity (FC) (Fang et al., 2013), seed-based FC (Fang et al., 2016), and independent component analysis (Fang et al., 2015) of resting-state functional magnetic resonance imaging (Rs-fMRI), our previous studies demonstrated that dysfunctions in the cerebellum and its output motor and prefrontal cortices circuits were associated with tremors and cognitive impairment in patients with ET. More recently, using local FC (Duan et al., 2021) and graph theory analyses (Li et al., 2021) of Rs-fMRI, we and Li et al. found that the cerebellar-prefrontal cortices circuits were also associated with patients with depressed ET. Moreover, a diffusion tensor imaging study (Sengul et al., 2019) showed that the microstructure changes in the amygdala, hippocampus, and caudate nucleus were observed in patients with depressed ET. However, due to traditionally mass univariate analyses, all these above studies could not be used to diagnose the individual patient with depressed ET and had difficulty in sensitively identifying the change in spatially distributed patterns of these Rs-fMRI data.

Recently, the application of multivariate pattern analysis (MVPA) to neuroimaging data has gained increasing attention. Compared with the traditional group-level univariate analysis, MVPA not only could extract stable and identifiable features from Rs-fMRI data to distinguish subjects at the individual level but also have the advantage of being more sensitive to subtle and spatially distributed information due to taking the intercorrelation between voxels into consideration (Pereira et al., 2009). This method has been used to identify individual patients with schizophrenia (Xiao et al., 2017), Parkinson's disease (PD) (Tang et al., 2018), and patients with major depression (Guo et al., 2020). To the best of our knowledge, no study has combined machine learning MVPA with Rs-fMRI to identify patients with depressed ET.

In this study, we explored whether voxel-wise global brain connectivity (GBC) mapping of Rs-fMRI combined with machine learning MVPA [i.e., multiclass and binary Gaussian process classification (GPC) and binary support vector machine (SVM)] could be used to identify patients with depressed ET from non-depressed ET, primary depression, and healthy controls (HCs). We expected that these classification models could achieve good accuracy, and these brain regions of the significant discriminative features would help to reveal the large-scale brain network pathogenesis of depression in patients with ET.

## Materials and Methods

### Subjects

Our subjects consisted of 41 (i.e., 26 males and 15 females) patients with depressed ET, 49 patients with non-depressed ET, 45 patients with primary depression, and 43 age- and sex-matched HCs. Each subject signed an informed consent form approved by the Ethics Committee of the First Affiliated Hospital of Chongqing Medical University (Chongqing, China), and this study was performed in accordance with the Declaration of Helsinki of the World Medical Association. Several subjects included in this study have been reported by our previous MRI study (Duan et al., 2021). All these subjects fulfilled the following criteria: (1) the patients with ET met the diagnosis of definite or probable ET according to the Movement Disorders Consensus Criteria (Deuschl et al., 1998), and all the patients with ET had annual follow-ups through the outpatient department or by telephone; (2) the patients with probable ET were followed-up for at least 3 years to confirm the diagnosis; (3) the patients with ET had an onset age between 18 and 55 years old, and patients with earlier or later onset were not included; (4) the patients with ET were not treated with any anti-ET or antidepressant medications, and the patients with primary depression were not treated with any antidepressant medications before the baseline fMRI scan (only the baseline fMRI scan data were used in this study); (5) the patients were without any apparent cognitive impairment [Mini-Mental State Examination (MMSE) scores > 24] and were right-handed; (6) the patients with ET presented with moderate or greater amplitude kinetic tremor (tremor rating ≥ 2 during at least three tests); (7) the patients with ET were without PD, dystonia, psychogenic tremor, thyroid disease, stroke, epilepsy, head injury, or any other neurological dysfunction; and (8) the patients with depressed ET and patients with primary depression met the diagnostic and statistical manual of mental disorders version four (DSM-IV) criteria (Cooper, 1995), i.e., all the patients had to have one or both of the two main symptoms (i.e., depressed mood, loss of interest, or pleasure) that had lasted for more than 2 weeks.

The depression severity of each patient was evaluated by the 17-item Hamilton Depression Rating Scale (HDRS-17), and all patients with a score of at least 7 points were considered depressive (Bobo et al., 2016). Tremor severity was assessed using the Fahn-Tolosa-Marin Tremor Rating Scale (TRS) (Fahn et al., 1988) and the Essential Tremor Rating Assessment Scale (TETRAS) (Elble et al., 2012). The Hamilton Anxiety Rating Scale (HARS-14) (Bruss et al., 1994) was used to assess the anxiety severity of all the participants. The MMSE was used to briefly assess cognitive function and to screen for dementia.

### MRI Data Acquisition

All MR images were acquired using a GE Signa Hdxt 3T scanner (General Electric Medical Systems, Waukesha, WI, USA) equipped with a standard 8-channel head coil. Foam padding and earplugs were used to minimize head motion and to reduce scanner noise. During RS-fMRI scanning, all subjects were told to relax, to remain still with their eyes closed, and to remain awake (which was immediately confirmed *via* post-scan debriefing). RS-fMRI data were acquired using an echo-planar imaging (EPI) pulse sequence with the following parameters: 33 axial slices, slice thickness/gap = 4.0/0 mm, matrix = 64 × 64, repetition time (TR) = 2000 ms, echo time (TE) = 40 ms, flip angle = 90°, field of view (FOV) = 240 × 240 mm, and a total of 240 volumes were obtained (duration = 8 min). High-resolution 3D T1-weighted images (TR = 8.3 ms, TE = 3.3 ms, flip angle = 15°, slice thickness/gap = 1.0/0 mm, FOV = 240 × 240 mm, and matrix = 256 × 192) and T2-weighted FLAIR images (TR = 8,000 ms, TE = 126 ms, TI = 1,500 ms, slice thickness/gap = 5.0/1.5 mm, FOV = 240 × 240 mm, and matrix = 256 × 192) were also acquired. We did not use the T2-weighted FLAIR images for data processing, but they were used for image evaluation and data quality assessment.

### Data Preprocessing

Data preprocessing was conducted using the DPARSFA toolbox version 2.2 (http://rfmri.org/DPARSF) on MATLAB (MathWorks Inc., Natick, MA, USA) platform as previously described, and the preprocessing steps were as follows: (1) removal of the first 10 time points. For scanner stabilization and the acclimation of subjects to the MR scanning environment, the first 10 volumes were discarded, and the remaining 230 time points were included in the subsequent data preprocessing; (2) slice timing correction. This was used to correct for a different acquisition time across slices in a volume; (3) realignment. This was used to realign the subsequent functional images to the first volume to correct for within-run head motions, resulting in six rigid-body head motion parameters. These parameters were employed to assess the head movement and ensure the quality of RS-fMRI data; (4) T1 segmentation and spatial normalization. 3D T1-weighted images were segmented into gray matter (GM), white matter (WM), and cerebrospinal fluid (CSF) probability maps using SPM DARTEL segmentation. All the GM, WM, and CSF images were resampled to 1.5 × 1.5 × 1.5 mm^3^, spatially normalized to the MNI space using both affine transformation and non-linear deformation, and later, resampled to 3 × 3 × 3 mm^3^ voxel resolution with RS-fMRI, and the deformation field was applied to the RS-fMRI data (before step 4, the 3D-T1 images were co-registered to the mean RS-fMRI data for each subject); (5) removal of six head motion parameters and the mean time series of global, WM, and CSF signals; and (6) detrending and filtering. These steps removed the extremely low-frequency drift and the high-frequency physiological noises. For detrending, we used first-order polynomial functions; and for filtering, we adopted band-pass filtering (0.01 Hz < *f* < 0.08 Hz) to the time series for each voxel. Additionally, the image quality met a mean framewise displacement (FD) head motionless than <0.3 mm, and scrubbing of head motion volumes <50% volumes (115 volumes) (note: we just calculated a scrubbing of head motion volumes, used the scrubbing of head motion volumes <50% volumes as quality control criterion, and did not perform scrubbing in data processing).

### Head Motion Control

Due to the intrinsic BOLD signal contaminated by head motion and non-neuronal physiological processes being the major obstacle in the analysis of Rs-fMRI data, we performed systematic tactics to deal with head motion. First, we regressed six head motion parameters including translational (i.e., x, y, and z axes) and rotational (i.e., pitch, yaw, and roll). Second, we regressed nuisance signals, such as WM and CSF, and also global signals. Third, we also dealt with the volume-to-volume head motion, also called FDs. Using mean FDpower > 0.3 mm as a threshold (not FDJenk), the scrubbing volumes and the maximal scrubbing volumes were counted in this study, and one-way ANOVA and *post-hoc t*-test were performed to explore whether these head parameters exist significant differences among the four groups. The results showed that the maximal scrubbing volumes were 27 volumes (27/230 = 11.73%) in this study. No significant difference in scrubbing volumes and the mean FDpower among the four groups was observed (scrubbing volumes: 13.6585 ± 6.3270, 13.6829 ± 5.5337, 13.6976 ± 5.1248, and 14.5556 ± 5.6510; mean FDpower: 0.086 ± 0.0404, 0.0757 ± 0.0404, 0.0749 ± 0.0430, and 0.089 ± 0.0612; *F* = 0.2630, *P* = 0.8520; *F* = 0.6450, and *P* = 0.5870). Finally, a Pearson correlation analysis was performed between the mean FD power values and GBC values of regions of interest (ROIs) in patients with depressed ET, no significant correlation was observed, and we also used the mean FDpower values as a covariate in the correlation analysis between GBC values of ROIs and HDRS-17 scores in patients with depressed ET.

### Calculation of Voxel-Wise GBC Mappings

We adopted degree centrality as a metric to calculate the voxel-wise GBC mappings as Zuo et al. (2012) and our previous studies (Wang et al., 2018) described. In brief, an individual Pearson correlation coefficient (*r*) matrix was obtained by computing the Pearson correlation coefficient between each pair of voxels in an SPM8 prior probabilistic brain GM template as the mask (54,326 voxels; voxel size: 3 × 3 × 3 mm^3^) from each subject, and the threshold for the Pearson correlation coefficient was set at *r* ≥ 0.25. To improve normality, correlation matrix of each individual was transformed into a *Z*-score matrix using Fisher's *r*-to-*z* and *Z* transformation. Then, the individual degree centrality mapping was formed, smoothed with a 6 × 6 × 6 mm^3^ full-width at half-maximum (FWHM) Gaussian smoothing kernel (the data preprocessing without smoothing), and selected for further analysis in this study.

### Machine Learning Classification: MVPA

The multiclass and binary GPC and binary SVM algorithms of MVPA were performed using PRoNTo version 2.1.1 (http://www.mlnl.cs.ucl.ac.uk/pronto/) on MATLAB (MathWorks Inc., Natick, MA, USA) platform, and we adopted four-class GPC to identify depressed ET, non-depressed ET, primary depression and HCs, and binary GPC and binary SVM for depressed ET vs. non-depressed ET, depressed ET vs. primary depression, depressed ET vs. HCs, and primary depression vs. HCs, based on their respective individual GBC mapping. In brief, these machine learning MVPA were composed of five main analysis modules, namely, data set specification, feature set selection, model specification, model estimation, and weight computation. In the data set specification and feature set selection, the individual GBC mapping was inputted into machine learning algorithms as features, and the mean DARTEL GM mask was used to exclude uninteresting features. In the model specification and model estimation, the features were mean-centered, and multiclass GPC, binary GPC, and binary SVM were used to test whether the individual GBC mapping could be used to discriminate these subjects. These subjects were divided into training and testing sets, and a leave-one-subject-out-cross-validation (LOSOCV) was used. We took the sensitivity, specificity, accuracy, balanced accuracy, total accuracy, positive predictive value, negative predictive value, and receiver operating characteristic (ROC) curve (only in binary GPC and binary SVM) into account to evaluate the performance of these classification models. We used the permutation test to assess the significance of the performance of these models and to locate the significant discriminative features. More specifically, we repeated the permutation cross-validation procedure test 1,000 times and counted how many times the value of these accuracy measures was equal to or higher than the correct one. The *p*-value was then calculated by dividing this number by the number of permutations (1,000). To locate the significant discriminative features, the contribution of each voxel to classification was calculated, the *P*-value of voxels was calculated by dividing this number by the number of permutations (1,000), then it was projected to generate the discriminative map, and the cluster size > 30 voxels was adopted.

### Correlation Analysis Between GBC Values of Significant Discriminative Features and Clinical Depression Severity

Finally, a univariate analysis was further used to investigate the correlations between the GBC values of significant discriminative features and the HDRS-17 scores in patients with depressed ET. In brief, these clusters of the significant discriminative features were defined as an ROI, we abstracted the mean GBC values of these ROIs, and a Pearson correlation analysis between the mean GBC values of these ROIs and the HDRS-17 scores of the patients with depressed ET was performed with Bonferroni multiple comparison corrections.

In addition, we also performed *post-hoc* analyses to evaluate the potential impact of HARS-14 scores and education year differences between primary depression and depressed ET on the results. A univariate two-sample *t*-test with the age, education years, and scores of the MMSE and HARS-14 as covariates was performed, and the significant discrimination features of primary depression vs. depressed ET were used as a mask (to consider that we only focused on whether these significant discrimination features were influenced by these above covariates, and we did not use a GM or whole-brain mask) with permutation (1,000) and threshold-free cluster enhancement (TFCE) multiple comparison correction (corrected *P* < 0.001). Meantime, in the univariate correlation analysis, we also used age, education years, and scores of the MMSE and HARS-14 as covariates and a partial Pearson correlation was performed.

## Results

### Demographic and Clinical Characteristics

Demographic and clinical information is shown in Table 1, and the age, education level, tremor of onset, and scores on TRS parts A and B, TRS part C, TETRAS, TETRAS-ADL, MMSE, HDRS-17, and HARS-14 in patients with depressed ET showed a normal distribution (*P* = 0.63, 0.12, 0.32, 0.66, 0.33, 0.45, 0.27, 0.06, 0.97, and 0.06, respectively), and the tremor duration in the patients with depressed ET showed a non-normal distribution (*P* = 0.035). Among these clinical data, a significant correlation was observed between TRS parts A and B and TETRAS and TRS part C and TETRAS-ADL (Pearson's: *r* = 0.61, *P* = 3.542E-5; *r* = 0.47, *P* = 0.003, respectively), and a marginally significant correlation was observed between HDRS-17 scores and education level (Pearson's: *r* = 0.27, *P* = 0.09) in patients with depressed ET.

**Table 1:** Demographic and clinical features of depressed ET, non-depressed ET, HCs, and DP.

**Measure**	**DET(41)**	**ET(49)**	**HCs(43)**	**DP(45)**	**Statistic**	***P-value***			
						**DET vs. ET**	**DET vs. HCs**	**DET vs. DP**	**DP vs. HCs**
**Demographic**									
Age	47.85 ± 15.66	46.16 ± 14.52	46.60 ± 13.45	45.20 ± 13.25	*F* = 0.50	0.37	0.47	0.24	0.64
Sex (male/female)	26:15	32:17	27:16	20:25	*x*^2^ = 6.54	0.85	0.95	0.80	0.09
Education (year)	14.73 ± 4.17	12.18 ± 3.95	12.28 ± 3.49	11.95 ± 4.40	*F* = 4.43	0.03	0.06	0.02	0.71
Handedness (R/L)	41:0	49:0	43:0	45:0	*x*^2^ = 0	1	1	1	1
**Clinical of psychology and cognitive**									
HDRS-17	18.98 ± 6.35	3.27 ± 1.60	2.09 ± 1.19	25.31 ± 7.12	*F* = 258.21	1e-6	1e-6	0.25	1e-6
MMSE	26.00 ± 1.40	26.67 ± 1.71	28.70 ± 1.35	25.67 ± 1.77	*F* = 31.99	0.05	1e-6	0.33	1e-6
HARS-14	7.56 ± 3.47	3.73 ± 1.90	2.02 ± 1.18	9.29 ± 4.93	*F* = 48.47	1e-6	1e-6	0.01	1e-6
**Clinical of tremor**									
Tremor of onset	34.93 ± 11.84	34.43 ± 10.16	NA	NA	*T* = 0.21	0.83	NA	NA	NA
Tremor duration	13.90 ± 9.40	12.69 ± 7.02	NA	NA	*T* = 0.70	0.49	NA	NA	NA
TRS-parts A&B	22.66 ± 7.28	20.90 ± 7.35	NA	NA	*T* = 1.14	0.26	NA	NA	NA
TRS-part C	14.05 ± 4.74	11.82 ± 5.80	NA	NA	*T* = 1.97	0.05	NA	NA	NA
TETRAS	18.32 ± 8.27	17.63 ± 6.75	NA	NA	*T* = 0.57	0.57	NA	NA	NA
TETRAS-ADL	21.39 ± 6.51	20.91 ± 5.66	NA	NA	*T* = 1.31	0.19	NA	NA	NA

*ET, essential tremor; DET, depressed ET; HCs, healthy controls; DP, primary depression; HDRS-17, 17-item Hamilton Depression Rating Scale; MMSE, Mini-Mental State Examination; HARS-14, 14-item Hamilton Anxiety Rating Scale; TRS, Fahn-Tolosa-Marin Tremor Rating Scale; TETRAS, Essential Tremor Rating Assessment Scale; TETRAS-ADL, Essential Tremor Rating Assessment Scale-Activities of Daily Living*.

### Multiclass GPC Classification

Using GBC mapping, the four-class GPC machine learning MVPA achieved a total accuracy of 40.45% and a balanced accuracy of 41.16% for the whole four classes of depressed ET, non-depressed ET, primary depression, and HCs, the accuracy for depressed ET, non-depressed ET, primary depression, and HCs was 70.73, 18.37, 75.56, and 0.00%, respectively, and statistically significant accuracy was *P* < 0.001, 0.922, 0.001, and 1.000, respectively (Supplementary Figure 1).

### Binary GPC and SVM Classification

**Figure 1:**
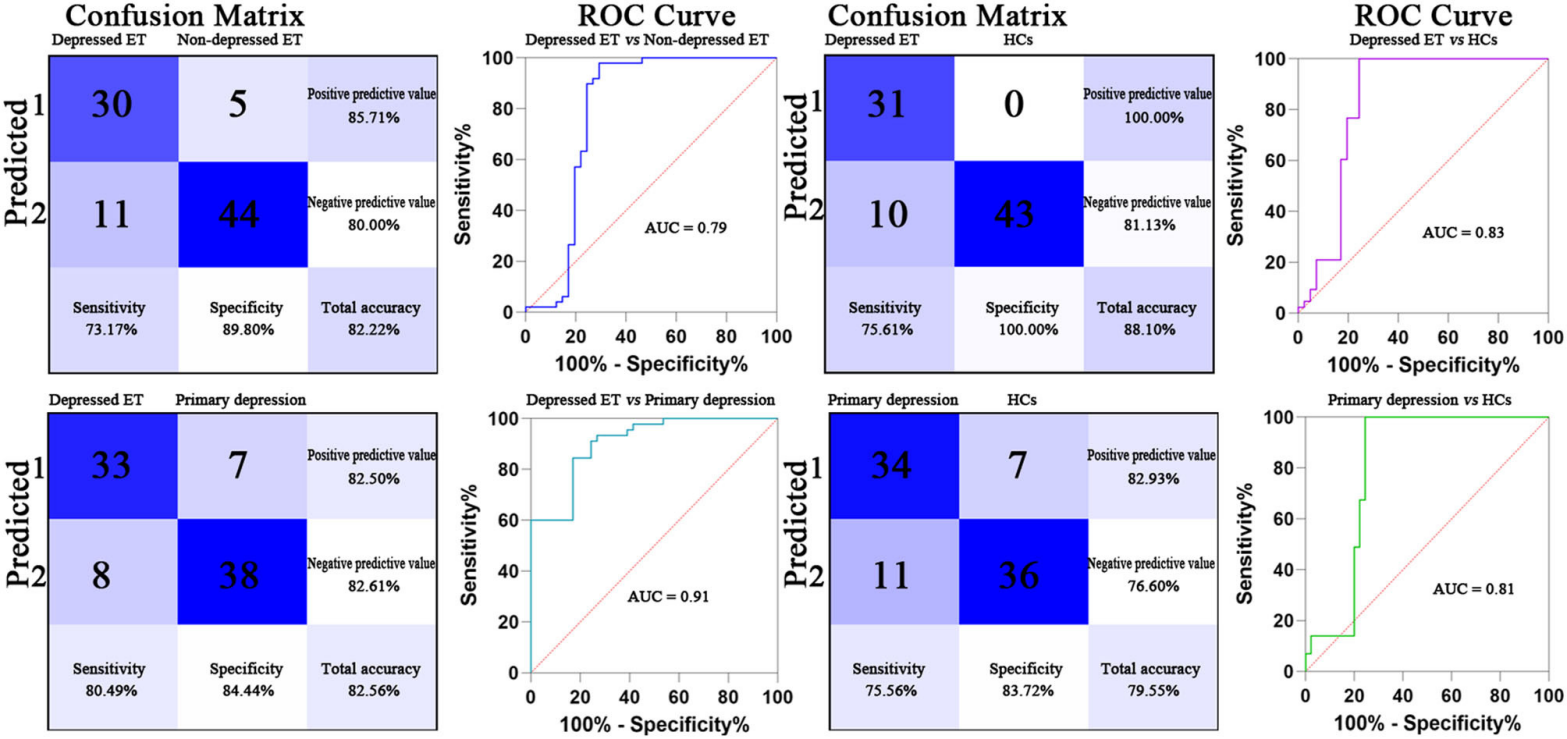
Confusion matrix and ROC curve of the binary SVM algorithm. ET, essential tremor; HCs, healthy controls; ROC, receiver operating characteristic; AUC, area under the curve; SVM, support vector machine.

Based on voxel-wise GBC mapping, these binary GPC and binary SVM machine learning MVPA were able to discriminate depressed ET vs. HCs, depressed ET vs. non-depressed ET, depressed ET vs. primary depression, and primary depression vs. HCs, and the results of SVM were better than GPC and the following presented with binary SVM results. Figure 1 shows the classification of depressed ET vs. HCs with a balanced accuracy of 87.80% and permutation test with statistically significant balanced accuracy, sensitivity, and specificity at *P* < 0.001; the classification of depressed ET vs. non-depressed ET with a balanced accuracy of 81.48% and permutation test with statistically significant balanced accuracy, sensitivity, and specificity at *P* < 0.001; the classification of depressed ET vs. primary depression with a balanced accuracy of 82.47% and permutation test with statistically significant balanced accuracy, sensitivity, and specificity at *P* < 0.001; and the classification of primary depression vs. HCs with a balanced accuracy of 79.64% and permutation test with statistically significant balanced accuracy, sensitivity, and specificity at *P* < 0.001.

**Figure 2:**
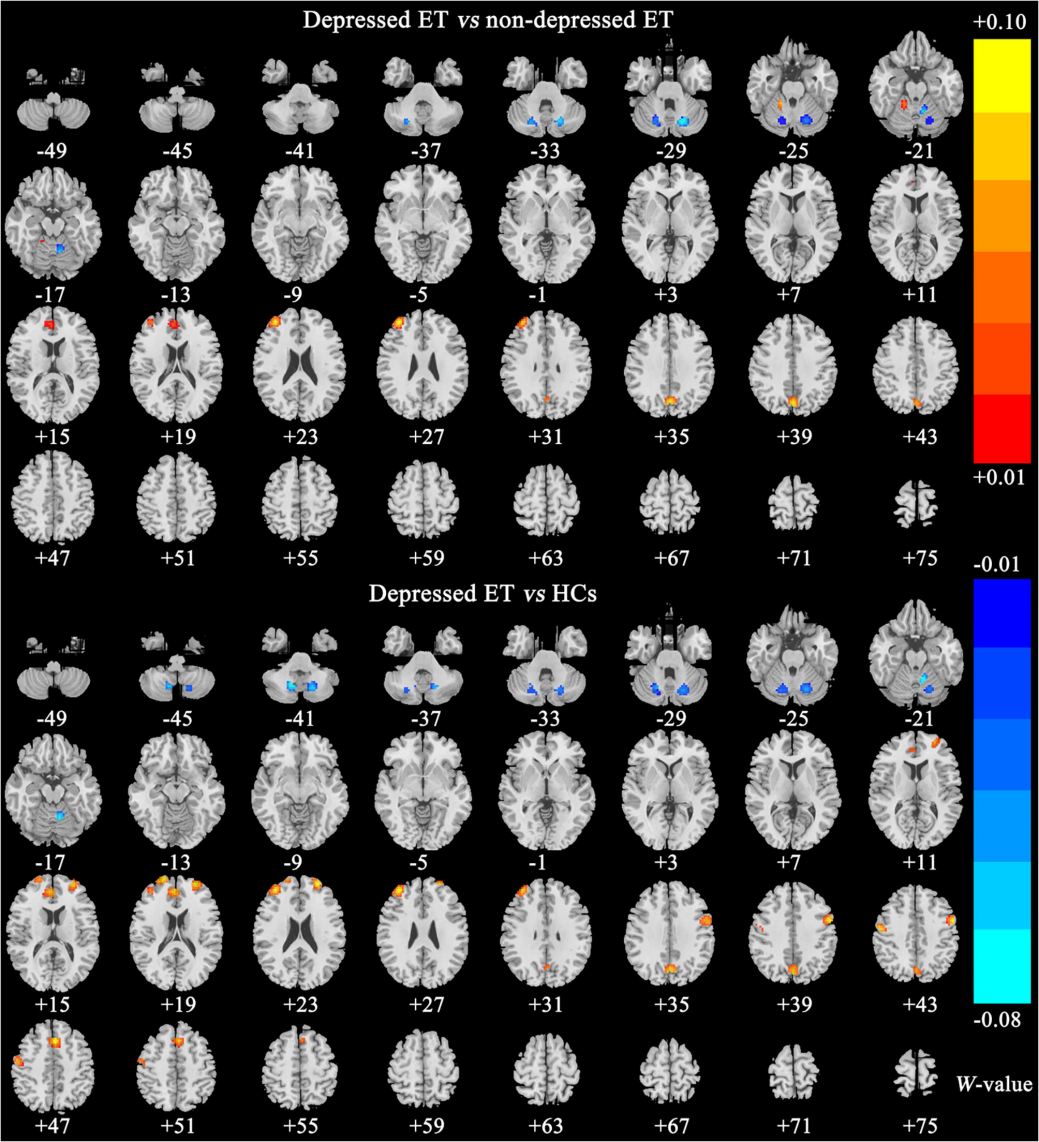
The brain regions of significant discriminative features in classification of depressed ET vs. non-depressed ET and depressed ET vs. HCs. ET, essential tremor; HCs, healthy controls.

**Figure 3:**
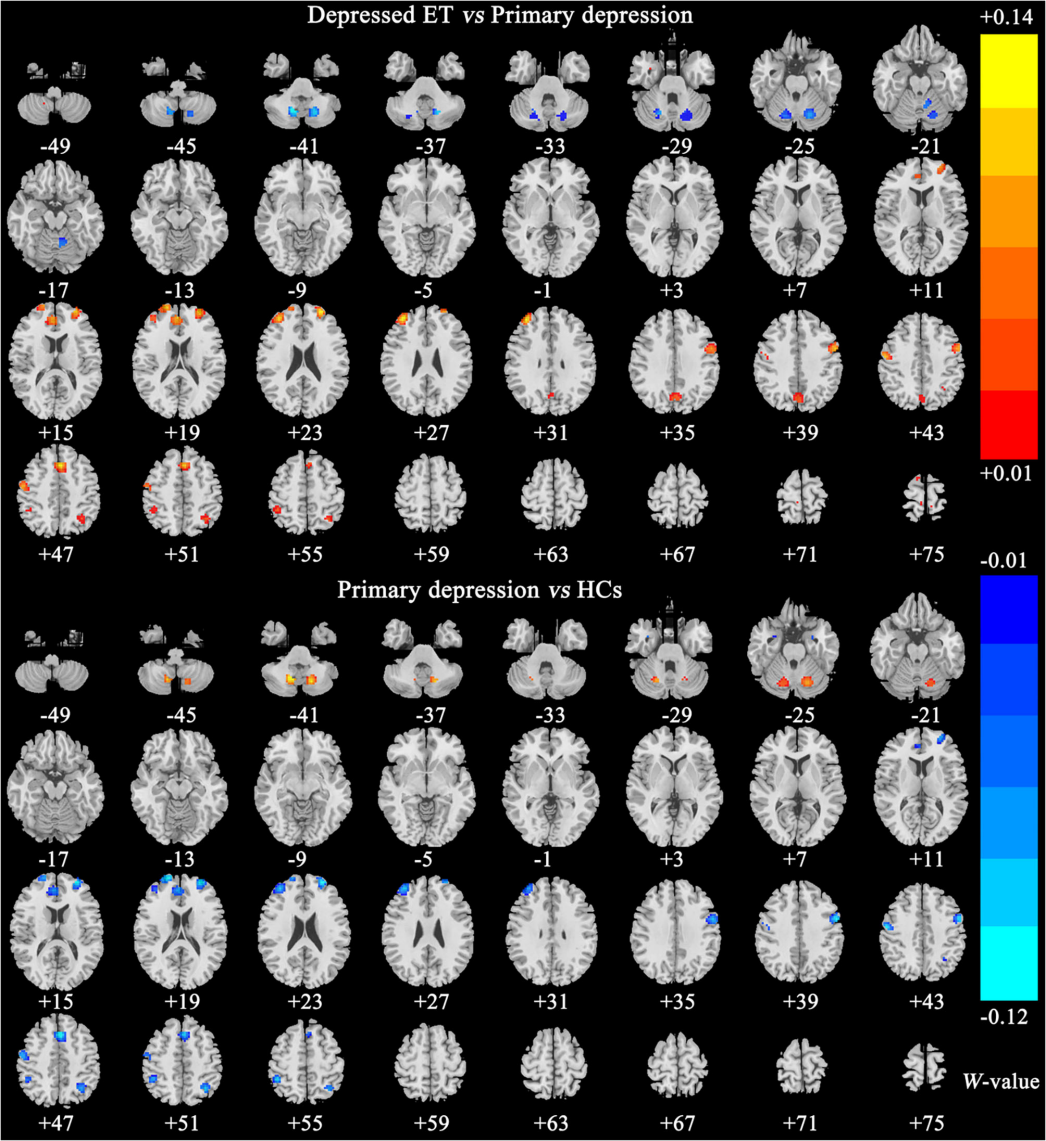
The brain regions of significant discriminative features in classification of depressed ET vs. primary depression, and primary depression vs. HCs. ET, essential tremor; HCs, healthy controls.

Figures 2, 3 show the corresponding significant discriminative features of these classifications with permutation test at *P* < 0.001. In depressed ET vs. HCs, positive (mean depressed ET > HCs) discriminative features located in the bilateral supplementary motor cortices, bilateral precentral cortices, bilateral anterior cingulate cortices, bilateral precuneus gyri, right middle frontal gyrus, and left superior frontal gyrus, and negative (mean depressed ET < HCs) discriminative features located in bilateral cerebellum VIII, bilateral cerebellum VI, and left cerebellum IV–V; in depressed ET vs. non-depressed ET, positive (mean depressed ET > non-depressed ET) discriminative features located in the bilateral anterior cingulate cortices, bilateral precuneus gyri, right middle frontal gyrus, and right cerebellum IV–V, and negative (mean depressed ET < non-depressed ET) discriminative features located in bilateral cerebellum VI and Crus I and left cerebellum IV–V; in depressed ET vs. primary depression, positive (mean depressed ET > primary depression) discriminative features located in the bilateral supplementary motor cortices, bilateral precentral cortices, bilateral precuneus gyri, bilateral middle frontal gyri, bilateral superior frontal gyri, bilateral anterior cingulate cortices, and bilateral inferior parietal lobules, and negative (mean depressed ET < primary depression) discriminative features located in bilateral cerebellum VIII, bilateral cerebellum VI and Crus I, and left cerebellum IV–V; and in primary depression vs. HCs, positive (mean primary depression > HCs) discriminative features located in the bilateral cerebellum VIII and bilateral cerebellum VI, and negative (mean primary depression < HCs) discriminative features located in bilateral supplementary motor cortices, bilateral precentral cortices, bilateral middle frontal gyri, bilateral superior frontal gyri, bilateral anterior cingulate cortices, bilateral inferior parietal lobules, and bilateral amygdale. In addition, the results of the univariate two-sample *t*-test with the age, education years, and scores of the MMSE and HARS-14 as covariates between primary depression and depressed ET were similar to the results of machine learning.

### Correlation Between GBC Values and HDRS-17 Scores

**Figure 4:**
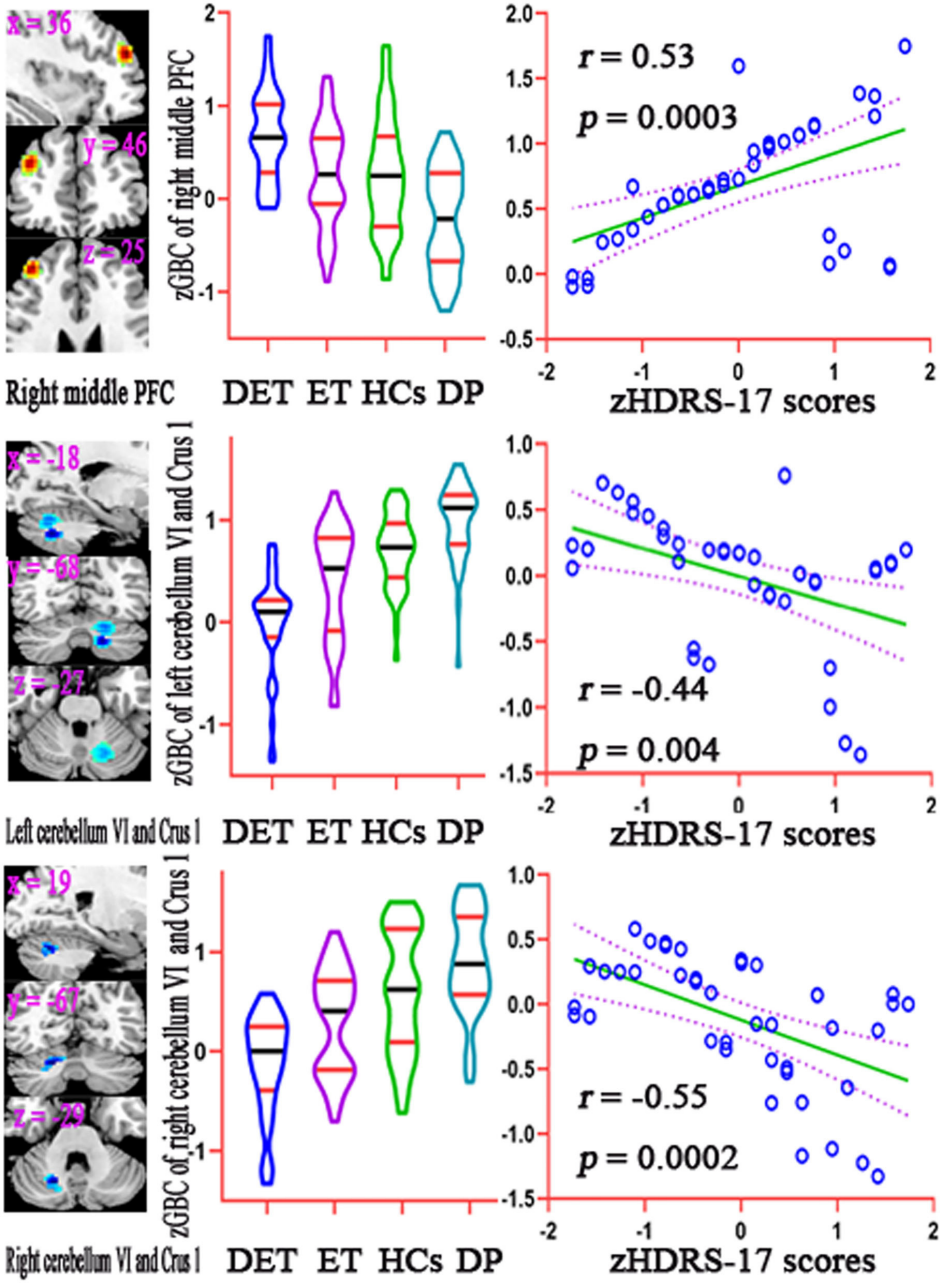
Results of correlation analysis between the GBC values of significant discriminative features and the HDRS-17 scores in patients with depressed ET. Bonferroni multiple comparison corrections, corrected *P* < 0.05/12 × (12–1)/2. Left: the cluster of the significant discriminative features. Middle: mean GBC values in the significant discriminative regions among DET, ET, HC, and DP groups. Right: the scatter plots for the correlation analysis in patients with depressed ET. GBC, global brain connectivity; PFC, prefrontal cortex; zGBC, z-transformed global brain connectivity; zHDRS-17 scores, z-transformed Hamilton Depression Rating Scale 17-item scores; DET, depressed essential tremor; ET, essential tremor; HCs, healthy controls; DP, primary depression; HDRS-17, 17-item Hamilton Depression Rating Scale.

Twelve clusters of the significant discriminative features (permutation test at *P* < 0.001) in the classification of depressed ET vs. HCs were observed, and 12 ROIs were further defined. A Pearson correlation analysis showed a significant positive correlation between GBC values of ROIs in the right middle prefrontal cortex and the HDRS-17 scores in patients with depressed ET, a significant negative correlation between GBC values of ROIs in right cerebellum VI and Crus 1 and the HDRS-17 scores in patients with depressed ET, and a marginally significant negative correlation between GBC values of ROIs in left cerebellum VI and Crus 1 and the HDRS-17 scores in the patients with depressed ET (Figure 4) (Bonferroni multiple comparison corrections, corrected *P* < 0.05/12 × (12 – 1)/2). In addition, the results of partial Pearson correlation were also similar.

## Discussion

Voxel-wise GBC mapping of Rs-fMRI combined with machine learning (i.e., multiclass GPC, binary GPC, and binary SVM) MVPA, the following three main findings were obtained: (1) although the four-class GPC achieved a totally poor classification, the four-class, binary GPC, and binary SVM could be used to discriminate depressed ET from non-depressed ET, primary depression, and HCs; (2) the significant discriminative features of these binary SVM and binary GPC MVPA mainly located in cerebellar-motor-prefrontal cortices circuits; and (3) the voxel-wise GBC values of significant discriminative features in right middle prefrontal gyrus and bilateral cerebellum VI and Crus 1 were correlated with clinical depression severity in patients with depressed ET.

### Machine Learning in Patients With ET

Before this study, very few studies combining clinical behavioral symptoms and signs, such as voice tremor (Suppa et al., 2021), motor task dysfunction (DeSimone et al., 2019), tremor parameters (Zheng et al., 2019), and balance and gait disorders (Moon et al., 2020), with machine learning approaches had achieved good classifications of ET vs. HCs, ET vs. PD, and ET vs. dystonia. However, these clinical behavioral symptoms were not more stable, direct, and precise as biological or physiological biomarkers, especially neuroimaging data, the most potential and easy to acquire biomarkers, and all above studies were binary classification approaches and did not involve the non-motor symptoms including depression in ET. More recently, only Benito-León et al. (2019) adopted MRI-derived brain volume and cortical thickness data to discriminate ET vs. orthostatic tremor and achieved a good classification. Therefore, this study was the first to combine neuroimaging data with machine learning algorithms to discriminate depressed ET from non-depressed ET, primary depression, and HCs and further to explore the large-scale neural network mechanisms for these diseases. Although the total and balanced accuracies for the four classes were poor (40.45 and 41.16%, respectively), the four-class classification had achieved a good accuracy for depressed ET (70.73%) and primary depression (75.56%) vs. the remaining three classes.

### Depression May Be a Primary Phenomenon in ET

It is still on the debate that whether depression is a primary or a secondary feature in ET. Based on the scores of Hamilton Depression Rating Scale being correlated with the scores of Fahn-Tolosa-Marin TRS parts A and B in patients with depressed ET, some studies (Chandran et al., 2012) highlighted that depression in ET was merely a secondary phenomenon in response to tremor severity. However, many studies (Louis et al., 2016; Huey et al., 2018) did not observe this correlated relationship. Consistent with these studies, this study failed to find any significant correlation between depression severity and other clinical characteristics, including the Fahn-Tolosa-Marin TRS parts A and B and TETRAS scores in the patients with depressed ET. Moreover, our findings showed that the multiclass GPC, binary GPC, and binary SVM MVPA could be used to discriminate depressed ET from non-depressed ET, primary depression, and HCs, these results showed that the spatially distributed patterns of voxel-wise GBC in patients with depressed ET were different from non-depressed ET, primary depression, and HCs, and the significant discriminative features in cerebellar-prefrontal cortices circuits were observed. Particularly, a growing body of study (Galts et al., 2019) suggested that the etiology of primary depression was extremely complicated, including monoaminergic and glutamatergic disturbances, neuroplasticity changes, hypothalamic-pituitary-adrenal axis dysfunction, neuroinflammation, and brain network reconfiguration. The interaction of molecular, biochemical, and functional changes was believed to contribute to the development of primary depression. However, previous ET studies failed to find the same dopaminergic neuron abnormality as observed in primary depression. Importantly, our findings of different connectivity patterns of cerebellar and its output cortices circuits between depressed ET and primary depression lent support to the view that there was different pathophysiology between depressed ET and primary depression. Although the HARS-14 scores and education years were significantly different between primary depression and depressed ET, the results of our additional analyses showed that the brain network change differences between primary depression and depressed ET were not related to the HARS-14 scores and education year differences, and these variables could not be confounding factors to hinder exploring the large-scale brain network changes between primary depression and depressed ET in this study. Therefore, due to the large-scale neural network spatially distributed pattern difference, we further suggested that depression in ET may be a primary manifestation of the disease rather than secondary to impaired motor functions and decreased quality of life due to tremor.

### The Cerebellar-Prefrontal Cortices Circuits Are a Key Pathogenesis Network Associated With Depression in Patients With ET

Recently, the adoption of Rs-fMRI to explore large-scale brain network changes gave advances in understanding the relationship between depression pathogenesis and cerebellar-prefrontal cortices circuits. Growing studies demonstrated that the changes of local, seed-based, and voxel-wise whole-brain FC in cerebellar-prefrontal cortices circuits were associated with depression symptoms in primary depression (Song et al., 2016) and movement disorders with depression, such as PD (Wang et al., 2018), Huntington's disease (Garcia-Gorro et al., 2019), and ET (Duan et al., 2021). Consistent with these studies, our findings demonstrated that voxel-wise GBC changes in cerebellar-prefrontal cortices circuits not only were the significant discriminative features in the classification of depressed ET from non-depressed ET, primary depression, and HCs but also voxel-wise GBC values in right middle prefrontal gyrus and bilateral cerebellum VI and Crus 1 were correlated with clinical depression severity in patients with depressed ET. So, we adhered that the cerebellar-prefrontal cortices circuits were a key pathogenesis network associated with depression in patients with ET. Meantime, previous studies (Gong and He, 2015) have found that connectivity disruption involved multiple brain regions in primary depression and is mainly located in the prefrontal-limbic-cerebellar pathway, including the prefrontal cortex, anterior cingulate cortex, precuneus gyrus, amygdala, caudate nucleus, and cerebellum. The discriminative regions identified in our patients with primary depression were similar to those observed in previous primary depression studies. Moreover, the voxel-wise GBC changes in cerebellar-prefrontal cortices circuits as common discriminative features in the classification of depressed ET vs. HCs and primary depression vs. HCs seemed that depressed ET and primary depression shared a similar cerebellar-prefrontal cortices circuits pathogenesis. However, in the classification of primary depression vs. HCs, extensive negative (mean primary depression < HCs) discriminative features in cerebral cortices including prefrontal cortices and positive discriminative features in the cerebellum were observed, and in the classification of depressed ET vs. HCs, these discriminative features were contrary to primary depression vs. HCs, and positive discriminative features in cerebral cortices including prefrontal cortices and negative discriminative features in the cerebellum were observed. Therefore, the spatially distributed patterns of voxel-wise GBC changes in depressed ET and primary depression were actually different, and these results further suggested that depressed ET and primary depression owned the different large-scale neural network pathogenesis.

In addition, previous studies (Louis and Vonsattel, 2008; Lin et al., 2014; Louis and Faust, 2020) from postmortem examinations have shown that only the cerebellum and brainstem existed clearly identifiable structural changes in the ET brain, and the other brain regions including prefrontal cortices, motor cortices, anterior cingulate cortices, and precuneus gyri did not have any neuropathology abnormalities. Combined previous postmortem examinations with our findings, we suggested that the cerebellum may play a key pathogenesis role in the cerebellar-prefrontal cortices circuits involved in ET with depression symptoms.

Besides, in another movement disorder, previous dystonia studies (Stamelou et al., 2012) have suggested that cortical-basal ganglia-cortical circuit dysfunction, especially the striatum, was a pathophysiological substrate of depression in dystonia. Unlike this, this study demonstrated that cerebellar-prefrontal cortices circuits dysfunction played an important role in the pathogenesis of depression in ET and emphasized the important role of the cerebellum and its output circuits in emotional regulation. Thus, our findings further indicated that there was a specificity of neuropathological features in movement disorders with depression.

### Limitations

Some limitations in this study need to be noted. First, although multiclass GPC, binary GPC, and binary SVM could be used to discriminate depressed ET from non-depressed ET, primary depression, and HCs, all these classification algorithms were supervised learning approaches, and the unsupervised learning algorithms based on a large sample may give more perfectly classification performance and even an achieved clinical diagnosis state. Second, we must admit that LOSOCV does have some shortcomings, including the possibility of result bias and longer computation time due to more data that are provided in the model training. Therefore, it is important to confirm our findings with the larger sample size and independent validation data in the future. Third, although based on our findings, we recommended that depression may be a primary phenomenon in ET, this study was cross-sectional, and the follow-up data may be more able to directly address these issues. Fourth, all the depressed ET, non-depressed ET, and primary depression patients were obtained from the outpatient department, our findings could not fully describe the depressive characteristics in these patients, and in the future, a population-based study will help to overcome this drawback. Finally, due to the absence of biological and pathogenic markers, the diagnosis of ET only relied on clinical phenomenology and neurological examinations, and the misdiagnosis is very common. However, all patients with ET had long follow-up periods to minimize the risk of misdiagnosis in this study.

## Conclusion

Using multiclass GPC, binary GPC, and binary SVM based on voxel-wise GBC mapping could identify depressed ET from non-depressed ET, primary depression, and HCs. The spatially distributed patterns of voxel-wise GBC changes in cerebellar-prefrontal cortices circuits not only played the significant discriminative features but also helped to understand the large-scale neural network pathogenesis underlying depression in patients with ET.

## Data Availability Statement

The raw data supporting the conclusions of this article will be made available by the authors, without undue reservation.

## Ethics Statement

The studies involving human participants were reviewed and approved by the Ethics Committee of the First Affiliated Hospital of Chongqing Medical University (Chongqing, China). The patients/participants provided their written informed consent to participate in this study.

## Author Contributions

YL: conception and execution of research project, design and execution of statistical analysis, and writing of the first draft of the manuscript. LT: conception and organization of research project, design and execution of statistical analysis, and writing of the first draft of the manuscript. HC and HW: execution of research project, execution and review and critique of statistical analysis, and review and critique of the manuscript. XiZ and XuZ: execution of research project, execution of statistical analysis, and review and critique of the manuscript. XD and ZF: execution of research project, review and critique of statistical analysis, and review and critique of the manuscript. QL and WH: execution of research project and review and critique of the manuscript. FL, JL, ZX, and JC: organization of research project, review and critique of statistical analysis, and review and critique of the manuscript. WF: conception and organization of research project, design, execution, review and critique of statistical analysis, and review and critique of the manuscript. All authors contributed to the article and approved the submitted version.

## Funding

This study was supported by the National Natural Science Foundation of China (NSFC: 81671663) and the Natural Science Foundation of Chongqing (NSFCQ: cstc2014jcyjA10047).

## Conflict of Interest

The authors declare that the research was conducted in the absence of any commercial or financial relationships that could be construed as a potential conflict of interest.

## Publisher's Note

All claims expressed in this article are solely those of the authors and do not necessarily represent those of their affiliated organizations, or those of the publisher, the editors and the reviewers. Any product that may be evaluated in this article, or claim that may be made by its manufacturer, is not guaranteed or endorsed by the publisher.
