# Supplementary material for: Identifying Depressed Essential Tremor Using Resting-State Voxel-Wise Global Brain Connectivity: A Multivariate Pattern Analysis
Source: Front Hum Neurosci. 2021 Oct 12;15:736155. doi: 10.3389/fnhum.2021.736155 (PMC8545862; doi:10.3389/fnhum.2021.736155)
Supplement: Supplementary file 1 [file Data_Sheet_1.pdf]

Figure S1. Confusion matrix of the four-class GPC algorithm.

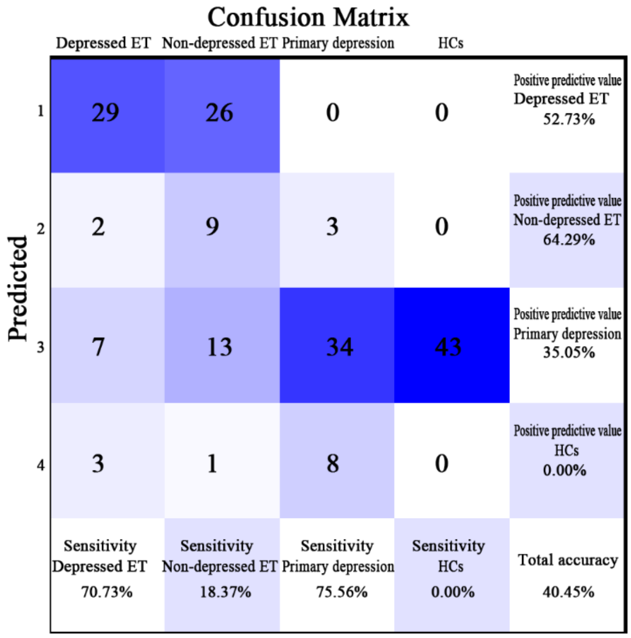

Table S1. The brain regions and peak MNI coordinates of significant discriminative features in classification of depressed ET *vs* non-depressed ET, depressed ET *vs* HCs, depressed ET *vs* primary depression and primary depression *vs* HCs.

Brain region	Size (voxels)	*W*-value	MNI coordinates *x*	*y*	*z*
**Depressed ET *vs* non-depressed ET**					
Cluster 1: cluster size: 167 voxels, peak MNI: 34 45 24, peak *W*-value: 0.10					
Right middle prefrontal gyrus	135	0.10	34	45	24
Cluster 2: cluster size: 134 voxels, peak MNI: -2 -72 39, peak *W*-value: 0.08					
Left precuneus gyrus	69	0.08	-2	-72	39
Right precuneus gyrus	51	0.07	4	-70	36
Cluster 3: cluster size: 149 voxels, peak MNI: 5 42 16, peak *W*-value: 0.05					
Right anterior cingulum cortex	73	0.05	5	42	16
Left anterior cingulum cortex	37	0.04	-1	42	16
Cluster 4: cluster size: 71 voxels, peak MNI: 19 -42 -25, peak *W*-value: 0.03					
Right cerebellum IV~V	63	0.04	19	-42	-25
Cluster 5: cluster size: 59 voxels, peak MNI: -10 -52 -19, peak *W*-value: -0.04					
Left cerebellum IV~V	51	-0.04	-10	-52	-19
Cluster 6: cluster size: 181voxels, peak MNI: -19 -71 -29, peak *W*-value: -0.06					
Left cerebellum crus 1	101	-0.06	-19	-71	-29
Left cerebellum VI	71	-0.05	-21	-68	-23
Cluster 7: cluster size: 173 voxels, peak MNI: 22 -71 -32, peak *W*-value: -0.07					
Right cerebellum crus 1	93	-0.07	22	-71	-32
Right cerebellum VI	61	-0.04	19	-67	-26
**Depressed ET *vs* HCs**					
Cluster 1: cluster size: 191 voxels, peak MNI: 36 46 25, peak *W*-value: 0.08					
Right middle prefrontal gyrus	174	0.08	36	46	25
Cluster 2: cluster size: 217 voxels, peak MNI: -24 55 23, peak *W*-value: 0.07					
Left superior prefrontal gyrus	91	0.07	-24	55	23
Left middle prefrontal gyrus	101	0.07	-28	52	18
Cluster 3: cluster size: 189 voxels, peak MNI: -2 22 49, peak *W*-value: 0.07					
Left supplementary motor cortex	111	0.07	-2	22	49
Right supplementary motor cortex	53	0.05	3	22	48
Cluster 4: cluster size: 115 voxels, peak MNI: -53 4 40, peak *W*-value: 0.07					
Left precentral cortex	104	0.07	-53	4	40
Cluster 5: cluster size: 113 voxels, peak MNI: 52 -8 46, peak *W*-value: 0.06					
Right precentral cortex	97	0.06	52	-8	46
Cluster 6: cluster size: 157 voxels, peak MNI: 5 41 16, peak *W*-value: 0.06					
Right anterior cingulum cortex	109	0.06	5	41	16
Left anterior cingulum cortex	41	0.05	2	45	18

Cluster 7: cluster size: 165 voxels, peak MNI: -2 -71 38, peak *W*-value: 0.05					
Left precuneus gyrus	87	0.05	-2	-71	38
Right precuneus gyrus	61	0.04	4	-69	37
Cluster 8: cluster size: 73 voxels, peak MNI: -10 -53 -22, peak *W*-value: -0.08					
Left cerebellum IV~V	67	-0.04	-10	-53	-22
Cluster 9: cluster size: 131 voxels, peak MNI: -17 -64 -39, peak *W*-value: -0.08					
Left cerebellum VIII	124	-0.08	-17	-64	-39
Cluster 10: cluster size: 117 voxels, peak MNI: 13 -64 -40, peak *W*-value: -0.07					
Right cerebellum VIII	107	0.07	13	-64	-40
Cluster 11: cluster size: 191 voxels, peak MNI: 19 -67 -29, peak *W*-value: -0.06					
Right cerebellum VI	114	-0.06	19	-67	-29
Right cerebellum crus 1	71	-0.03	19	-70	-30
Cluster 12: cluster size: 177 voxels, peak MNI: -18 -68 -27, peak *W*-value: -0.05					
Left cerebellum crus 1	101	-0.05	-18	-68	-27
Left cerebellum VI	64	-0.03	-19	-67	-23
**Depressed ET *vs* primary depression**					
Cluster 1: cluster size: 189 voxels, peak MNI: 37 46 27, peak *W*-value: 0.14					
Right middle prefrontal gyrus	176	0.14	37	46	27
Cluster 2: cluster size: 223 voxels, peak MNI: -25 58 23, peak *W*-value: 0.11					
Left superior prefrontal gyrus	127	0.11	-25	58	23
Left middle prefrontal gyrus	59	0.09	-29	53	16
Cluster 3: cluster size: 197 voxels, peak MNI: -2 22 48, peak *W*-value: 0.10					
Left supplementary motor cortex	117	0.10	-2	22	48
Right supplementary motor cortex	47	0.06	4	23	48
Cluster 4: cluster size: 133 voxels, peak MNI: -55 2 39, peak *W*-value: 0.07					
Right precentral cortex	113	0.07	-55	2	39
Cluster 5: cluster size: 119 voxels, peak MNI: 53 -9 46, peak *W*-value: 0.07					
Left precentral cortex	107	4.05	-2	-58	35
Cluster 6: cluster size: 153 voxels, peak MNI: 5 43 16, peak *W*-value: 0.06					
Right anterior cingulum cortex	104	0.06	5	43	16
Left anterior cingulum cortex	43	0.05	2	45	14
Cluster 7: cluster size: 78 voxels, peak MNI: -36 -61 55, peak *W*-value: 0.03					
Left inferior parietal lobules	64	0.03	-36	-61	55
Cluster 8: cluster size: 119 voxels, peak MNI: -2 -69 36, peak *W*-value: 0.03					
Left precuneus gyrus	67	0.03	-2	-69	36
Right precuneus gyrus	51		1	-68	36
Cluster 9: cluster size: 51 voxels, peak MNI: 42 -44 53, peak *W*-value: 0.02					
Right inferior parietal lobules	43	0.02	42	-44	53
Cluster 10: cluster size: 44 voxels, peak MNI: -29 -1 -26, peak *W*-value: 0.02					
Left amygdale	35	0.02	-29	-1	-26
Cluster 11: cluster size: 40 voxels, peak MNI: 29 0 -26, peak *W*-value: 0.02					

Right amygdale	31	0.02	29	0	-26
Cluster 12: cluster size:193 voxels, peak MNI: -19 -62 -41, peak *W*-value: -0.12					
Left cerebellum VIII	171	-0.12	-19	-62	-41
Cluster 13: cluster size:167 voxels, peak MNI: 13 -64 -41, peak *W*-value: -0.11					
Right cerebellum VIII	149	-0.11	13	-64	-41
Cluster 14: cluster size:179 voxels, peak MNI: 19 -66 -28, peak *W*-value: -0.07					
Right cerebellum VI	131	-0.07	19	-66	-28
Right cerebellum crus 1	38	-0.02	22	-71	-33
Cluster 15: cluster size:169 voxels, peak MNI: 19 -66 -28, peak *W*-value: -0.05					
Left cerebellum VI	114	-0.05	-22	-69	-25
Left cerebellum crus 1	50	-0.03	-19	-68	-29
Cluster 16: cluster size:87 voxels, peak MNI: -11 -54 -17, peak *W*-value: -0.04					
Left cerebellum IV~V	76	-0.04	-11	-54	-17
**Primary depression *vs* HCs**					
Cluster 1: cluster size: 149 voxels, peak MNI: 11 -63 -41, peak *W*-value: 0.07					
Right cerebellum VIII	129	0.07	11	-63	-41
Cluster 2: cluster size: 133 voxels, peak MNI: -18 -66 -40, peak *W*-value: 0.07					
Left cerebellum VIII	119	0.07	-18	-66	-40
Cluster 3: cluster size: 142 voxels, peak MNI: -19 -68 -24, peak *W*-value: 0.05					
Left cerebellum VI	117	0.05	-19	-68	-24
Cluster 4: cluster size: 127 voxels, peak MNI: 18 -68 28, peak *W*-value: 0.04					
Right cerebellum VI	121	0.04	18	-68	-28
Cluster 5: cluster size: 131 voxels, peak MNI: 20 62 21, peak *W*-value: -0.08					
Right superior prefrontal gyrus	11	-0.08	20	62	21
Cluster 6: cluster size: 249 voxels, peak MNI: -27 56 21, peak *W*-value: -0.07					
Left superior prefrontal gyrus	134	-0.07	-27	56	21
Left middle prefrontal gyrus	78	-0.06	-26	55	17
Cluster 7: cluster size: 107 voxels, peak MNI: 36 -47 24, peak *W*-value: -0.06					
Right middle prefrontal gyrus	91	-0.06	36	47	24
Cluster 8: cluster size: 159 voxels, peak MNI: -2 19 48, peak *W*-value: -0.06					
Left supplementary motor cortex	87	-0.06	-2	19	48
Right supplementary motor cortex	57	-0.05	3	23	48
Cluster 9: cluster size: 115 voxels, peak MNI: 53 -7 44, peak *W*-value: -0.05					
Right precentral cortex	112	-0.05	53	-7	44
Cluster 10: cluster size: 101 voxels, peak MNI: -53 5 39, peak *W*-value: -0.04					
Left precentral cortex	97	-0.04	-53	5	39
Cluster 11: cluster size: 87 voxels, peak MNI: -33 -58 51, peak *W*-value: -0.04					
Left inferior parietal lobules	71	-0.04	-33	-58	51
Cluster 12: cluster size: 67 voxels, peak MNI: 43 -44 51, peak *W*-value: -0.03					
Right inferior parietal lobules	54	-0.03	43	-44	51
Cluster 13 cluster size: 51 voxels, peak MNI: -28 0 -26, peak *W*-value: -0.03					

Left amygdale	37	-0.03	-28	0	-26
Cluster 14 cluster size: 49 voxels, peak MNI: 43 -44 51, peak *W*-value: -0.03					
Right amygdale	35	-0.03	31	2	-26
